# Modern fertility awareness methods: wrist wearables capture the changes in temperature associated with the menstrual cycle

**DOI:** 10.1042/BSR20171279

**Published:** 2018-11-30

**Authors:** Mohaned Shilaih, Brianna M. Goodale, Lisa Falco, Florian Kübler, Valerie De Clerck, Brigitte Leeners

**Affiliations:** 1 Clinic for Reproductive Endocrinology, University Hospital, Zurich, Switzerland; 2 Ava AG, Zurich, Switzerland

**Keywords:** Basal body temperature, Fertility awareness methods, Menstrual cycle, Wrist skin temperature

## Abstract

Core and peripheral body temperatures are affected by changes in reproductive hormones during the menstrual cycle. Women worldwide use the basal body temperature (BBT) method to aid and prevent conception. However, prior research suggests that taking one’s daily temperature can prove inconvenient and subject to environmental factors. We investigate whether a more automatic, non-invasive temperature measurement system can detect changes in temperature across the menstrual cycle. We examined how wrist skin temperature (WST), measured with wearable sensors, correlates with urinary tests of ovulation and may serve as a new method of fertility tracking. One hundred and thirty-six eumenorrheic, non-pregnant women participated in an observational study. Participants wore WST biosensors during sleep and reported their daily activities. An at-home luteinizing hormone (LH) test was used to confirm ovulation. WST was recorded across 437 cycles (mean cycles/participant = 3.21, S.D. = 2.25). We tested the relationship between the fertile window and WST temperature shifts, using the BBT three-over-six rule. A sustained 3-day temperature shift was observed in 357/437 cycles (82%), with the lowest cycle temperature occurring in the fertile window 41% of the time. Most temporal shifts (307/357, 86%) occurred on ovulation day (OV) or later. The average early-luteal phase temperature was 0.33°C higher than in the fertile window. Menstrual cycle changes in WST were impervious to lifestyle factors, like having sex, alcohol, or eating prior to bed, that, in prior work, have been shown to obfuscate BBT readings. Although currently costlier than BBT, the present study suggests that WST could be a promising, convenient parameter for future multiparameter fertility awareness methods.

## Introduction

The biphasic basal body temperature (BBT) rhythm during the menstrual cycle has been reported and studied since the early 1900s [1], with the first observational study taking place in the 1960s [2]. Defined as the core body temperature during the body’s resting state, BBT is usually estimated by measuring one’s oral, rectal, or vaginal temperature immediately upon awakening and prior to any physical activity [3]. For most women, the BBT fluctuates in response to hormonal variations across the menstrual cycle. A woman’s BBT reaches its lowest point (*nadir*) in a given cycle around her fertile window, just prior to ovulation and corresponding to a peak in estrogen [4]. Prior work suggests that sperms can survive in the female genital tract for up to 6 days, with higher probability of conception occurring closer to ovulation [5–7]. Thus, we define the fertile window or the time frame in which conception can occur, as the 5 days leading up to ovulation and the day of ovulation itself. The probability of conception again drops sharply after ovulation, suggesting oocytes can only survive for 12–24 h without fertilization [6,8–10]. Thus, a dip in BBT may indicate imminent ovulation; after ovulation occurs, a woman’s BBT typically increases as progesterone levels rise [11,12]. BBT constitutes a retrospective indication of the fertile window, as one cannot identify a cycle’s nadir until the subsequent post-ovulation temperature rise [13].

Prior research suggests that reproductive hormones are largely responsible for this biphasic shift in temperature. Estrogen, progesterone, and testosterone act directly on the warm-sensitive and cold-sensitive neurones of the preoptic anterior hypothalamus [14,15], pointing to their involvement in thermoregulation. Further, multiple studies have demonstrated that estrogen lowers the core body temperature [16–20]; it, along with testosterone, both inhibit the body’s heat-retaining mechanisms and accelerate the body’s heat loss mechanisms. In contrast, progesterone has the opposite chemical effect, thus increasing BBT post-ovulation [15,18].

Tracking one’s BBT as a means of natural family planning appeals to many women. The start-up cost for participating has been historically cheap, requiring only the purchase of a thermometer [21,22]. A woman then takes her temperature each morning upon waking, recording it either electronically with an app or by hand on graph paper so that, for a given cycle, she has a chart of how her temperature has changed over time. Ideally, her temperature is taken at the same time everyday so as to minimize unintended variance due to circadian temperature shifts [23–25]. In clinical settings, BBT users are often encouraged to follow the ‘three-over-six’ rule to determine the start of their fertile window [2]; the three-over-six rule suggests an upward trend in temperature when, for the first time in a given cycle, three consecutive daily readings are higher than the six preceding daily temperatures [2,26]. Some practitioners will still count this as the start of the fertile window if only five of the six preceding days are lower in temperature [27]. Although not an exact indicator of ovulation [28], upward shifts in BBT align with a woman’s periovulatory and fertile window in most cycles [21,22,29]. The relative low cost of BBT and the ability to take one’s temperature at home may attract many women to BBT as a fertility tracking method.

While BBT requires little financial investment, its accuracy depends largely on dependable usage and remains open to interpretation error. As many researchers have noted, traditional BBT measurement cannot prospectively predict a woman’s fertile window; it can only indicate retrospectively whether ovulation has occurred [21,30,31]. Additionally, the method requires individuals to accurately read and chart their temperatures. Most women’s BBT shifts only 0.28–0.56°C [22,30]; thus, accurate measurement requires a sensitive thermometer. Reading one’s graph provides another opportunity to introduce unwanted bias into the process. If patients misinterpret their charts, they may wrongly identify their fertile window and thus reduce the method’s efficacy [30,31]. For this reason, it is recommended that women or couples interested in BBT attend training at a physician’s office [22]. Environmental factors, including sleeping in late, traveling across time zones, or alcohol consumption, can affect one’s BBT, creating additional unaccounted for variance in temperature fluctuations [30]. Finally, BBT may prove inconvenient for some women [21]; it requires a high level of patient compliance [22], with women having to wake up at the same time daily and meticulously chart their temperatures. Failure to do so can decrease the woman’s ability to correctly identify a biphasic shift in BBT when it occurs [32].

### Alternative fertility testing methods

BBT does not constitute the only method for determining the fertile window, however. Some women opt for use-at-home urine luteinizing hormone (LH) kits. Another hormone that varies across the menstrual cycle, LH rises sharply in most women anywhere from 16 to 48 h prior to ovulation [21]. This increase in LH can be detected in urine samples; specifically, LH test strips contain antibodies that bind to any LH molecules present in the urine. When the level of urinary LH is high enough, these bound complexes lead the test strip to change its color [22]. LH test kits range in simplicity, from single strips where users have to interpret the darkness of the LH presence line *vis-à-vis* a control line (e.g. Wondfo strips) to digital readers which, when fed the test strip, will electronically indicate ovulation status (e.g. ClearBlue Digital Ovulation kit). Women using the LH method often begin testing their urine on day 6 of their menstrual cycle and will continue to test for 5–9 days or until obtaining a positive indication of ovulation [33].

LH test kits present several potential advantages over BBT. First, they constitute a prospective measure of the fertile window; because they can detect the rise in LH prior to ovulation, obtaining positive LH test results allow a woman to know that she has 2–3 days of her fertile window left [21]. Second, LH testing is non-invasive and highly accurate with digital readers rendering the interpretation of the fertile window less prone to human error [22]. Whereas the BBT nadir aligns with the day of ovulation in only 43% of the cycles in fertile women [13], LH tests have been found to accurately predict ovulation in 90–100% of cycles [22]. The test strips can be commonly found over-the-counter, with a pack of individual test strips costing only a few dollars [21,22]. For some women, the method’s prospective nature and increased accuracy in identifying ovulation can make urinary LH testing a more favorable alternative to BBT.

However, urinary LH testing is not without its disadvantages. Whereas BBT thermometers constitute a one-time purchase, having to buy test strips for LH test kits may become expensive over numerous cycles [22]. Furthermore, the LH surge can still be missed by women, even those who test daily [22]. Additionally, even in those women who do manage to catch their LH surge, LH test kits can only indicate at most half of their fertile window; the days with the highest probability of conception often occur prior to a detectable LH surge [21,22]. This could explain why, despite LH test’s greater accuracy in detecting pending ovulation, studies have shown no difference in conception rates for women using the BBT or LH test method to track fertility [34].

Recent advances in technology have sought to improve upon the disadvantages of traditional BBT temping that make alternatives like LH testing more appealing. Multiple wearable devices have been developed in the past 10 years, claiming that they can estimate the day of ovulation by measuring temperature at various points in the body. For example, Tempdrop^i^ and Ava^ii^ measure skin temperature from the armpit and the wrist, respectively, while Yonoiii is an eardrop with a built-in thermometer that aims to estimate core body temperature. Less invasive than traditional thermometers, these wearable devices rest on the surface of the skin and record temperature readings every few seconds to every few minutes during sleep throughout the night. They synchronize to a phone application which then automatically records and charts a woman’s temperature, notifying her when the algorithm has detected a statistically significant upshift suggestive of the fertile window.

To date, few studies have considered the accuracy of new, wearable technology in predicting BBT shifts. There is some empirical precedence to suggest skin temperature—and not just oral or rectal temperature—may fluctuate in response to changes in the menstrual phase. Prior research, relying on skin sensors worn during a laboratory visit or at home, have shown an increase in skin temperature during the luteal phase in line with traditional BBT patterns [35]. However, past studies used multiple sensors across both waking and sleeping hours; to date, none have considered whether wrist skin temperature (WST) measured during sleep alone can adequately predict menstrual phase changes.

With current technology, we can now empirically address many of the disadvantages affecting traditional BBT compliance and accuracy. Continuous recording of WST, for example, provides a more robust method of estimating body temperature in comparison with BBT and other methods depending on self-measurement [35]. We propose conducting these measurements during sleep to capture the complete resting state and the minimum core body temperature known to occur then [3,36–40]. Moreover, how WST correlates with the different phases of the menstrual cycle has not been yet elucidated. Furthermore, we examine how factors known to confound BBT measurements affect WST. If shown to be accurate, wrist-wearable temperature sensors could comprise a reliable, user-friendly, and non-invasive alternative to traditional BBT [3,32–36].

### Aim of the present study

The aim of the present study is to evaluate whether WST patterns correlate with the different phases of the menstrual cycle when measured continuously during sleep. In addition, we will examine the agreement between urine ovulation detection kits and classical BBT methods applied to WST. Finally, we evaluate whether WST is impervious to environmental factors known to skew traditional BBT measurements.

## Materials and methods

### Participants

One hundred and ninety-four participants were recruited for an observational clinical study at the Department of Reproductive Endocrinology at the University Hospital Zurich, Switzerland. To be invited to participate in the study, women could not: be pregnant; have any known health-related issues; be taking medications known to affect the menstrual cycle; frequently fly across time zones; or have sleeping disorders. Further inclusion criteria were: between 20 and 40 years and regular menstrual cycles (self-reported length between 24 and 36 days). All study participants signed written informed consents. The ethical commission of the canton of Zurich, Switzerland approved the study protocol (approval number: KEK 170404) and the study was conducted accordingly.

### Study protocol

The participants measured their temperatures nightly during sleep using the Ava bracelet (Ava AG, Zürich, Switzerland), registered with the United States Food and Drug Administration as a fertility aid device. The Ava bracelet measures several physiological parameters including WST. Study participants were instructed to wear the bracelet on the dorsal side of the wrist while sleeping and on the same arm for the study’s duration.

Ovulation day (OV) was estimated using a urine LH home-ovulation test (Clearblue Digital Ovulation kit), which has been shown to have high concordance with ultrasound determination of ovulation (90–100% accurate [41]). Beginning 5 days after the onset of menses and extending through confirmed ovulation, women were requested to perform the LH test each morning at home. The LH test shows a smiling face indicating ‘peak fertility’, which in turn corresponds to OV-1 in most cases [42]. All participants were instructed on the usage of the tests by a trained nurse.

In addition to urinary tests and wearing the temperature-tracking bracelet nightly, participants also completed daily electronic diaries related to their activities and food consumption. Prior research on BBT tracking using traditional methods has found that consuming meals [43,44], drinking coffee [42], drinking alcohol [45], engaging in sexual intercourse, or heavy exercise [46] in the 3 h preceding sleep can significantly affect next-morning’s BBT; these confounders may mask or obscure temporal shifts caused by the menstrual cycle and of primary interest to the user. To test these activities’ effects on WST, we had women record their participation or consumption in their daily diaries. In addition to the above factors, women also reported spotting (defined as any bleeding occurring outside the menstrual phase). A more detailed description of the covariates and their measurement units can be found in our previously published paper [47,48], which shares similar methodology but a different outcome of interest.

The different phases of the menstrual cycle were labeled as follows: the **menstrual phase**, beginning with menses and lasting 5 days; the **follicular phase**, beginning with the first day post-menses and lasting through OV −6; the **fertile phase**, beginning with OV −5 and lasting through OV; the **early-luteal phase**, beginning with OV +1 and lasting through OV +7; and, the **late-luteal phase**, beginning with OV +8 and lasting through the day prior to menses’ onset.

### Data collection and data processing

All data processing and analysis were performed in R (v3.3.1) and Python 3.5. The Ava bracelet continuously recorded WST, providing one measurement every 10 s. To avoid variation induced by the initial drop in body temperature at the onset of sleep and subsequent rise prior to waking [49,50], the first 90 and the last 30 min of each night’s data were excluded. Consistent with best practices in non-parametric modeling and to remove artificial fluctuations due to the measurement tool [51,52], temperature data were logical regression smoothed (LOESS) before statistical analysis. The 99th (stable maxima) was chosen out of several percentiles (10, 50, 90%) to assess the correlation of WST with the different menstrual phases. There was no significant difference in a mixed effects model fit comparing data from the 99th percentile to data from the 50th or 90th percentiles, as assessed by pairwise log-likelihood tests (all *P*>0.05). The data from the 99th percentile was a significantly better fit than data from the 10th percentile (χ^2^ (15) = 11717, *P*<0.0001); thus, we chose to analyze the former percentile.

Consistent with the three-over-six rule’s underlying theory, we marked a shift in skin temperature when a woman’s WST rose at least 0.2°C above at least five of the preceding six days and stayed elevated for a minimum of three consecutive days [51,53,54]. Such an upward shift is useful in retrospectively confirming the occurrence of ovulation and the potential end of the fertile window. As temperature nadir was demonstrated in past BBT studies to be a prospective marker of ovulation [55], we also analyzed our WST results to see if a similar minimum temperature occurred approximating ovulation.

We used linear mixed effects models with random intercepts and random slopes to assess the association between WST and menstrual phases. Such models allow for the modeling of repeated measurements, accounting for correlated intra-individual and intracycle observations [56]. Because most participants reported multiple cycles and each cycle had its own phase shift, we analyzed our data using cross-classified models; we specified that cycle numbers were nested within individuals and the phases were nested within a cycle. Potential covariates previously shown to impact BBT were collected from participants’ daily diary reports and assessed for their effect on WST across the menstrual cycle using similar multilevel, cross-classified models as described above. Significant covariates were included in the final multivariate, multilevel model based on previously reported or potential clinical and practical relevance. Where appropriate, mean WST and its S.D. are reported in text. For each model, unstandardized b-coefficients and their S.E.M. are reported in Tables 1’3.

**Table 1:** Multilevel, linear mixed model of the relationship between menstrual phase and sleeping WST

	Unstandardized b-coefficient	S.E.M.
**Intercept**	35.06	0.10
**Cycle phase**		
Menstrual	Reference	Reference
Follicular	−0.01	0.04
Fertile	−0.13*	0.02
Early-luteal	0.24*	0.02
Late-luteal	0.37*	0.03

*refers to *P*<0.001.

## Results

### Descriptive statistics of the study population

Overall, 793 menstrual cycles were recorded across 194 study participants. In keeping with recommendations for best performance in fertility prediction algorithms [30,42–45], we excluded data from 186 cycles where participants reported measurements and synchronized their WST to the app for less than 80% of the days between the fertile window and the early-luteal phase. This left a reduced sample of 603 cycles across 159 participants. We also restricted our sample to participants with a confirmed ovulation, as detected via LH test; while an interesting extension of wearable technology, tracking potential anovulatory cycles is beyond the scope of the current study. Removing 170 cycles without confirmed LH surges left us with a final sample of 437 cycles across 136 participants; the average number of cycles per user in our sample was 3.21 (S.D. = 2.25). Participants had an average age of 33.66 years (S.D. = 3.86) and an average BMI of 22.97 kg/m^2^ (S.D. = 3.68). The average cycle length for the final sample was 28.84 days (S.D. = 7.02).

### Skin temperature rhythm during the menstrual cycle

**Figure 1:**
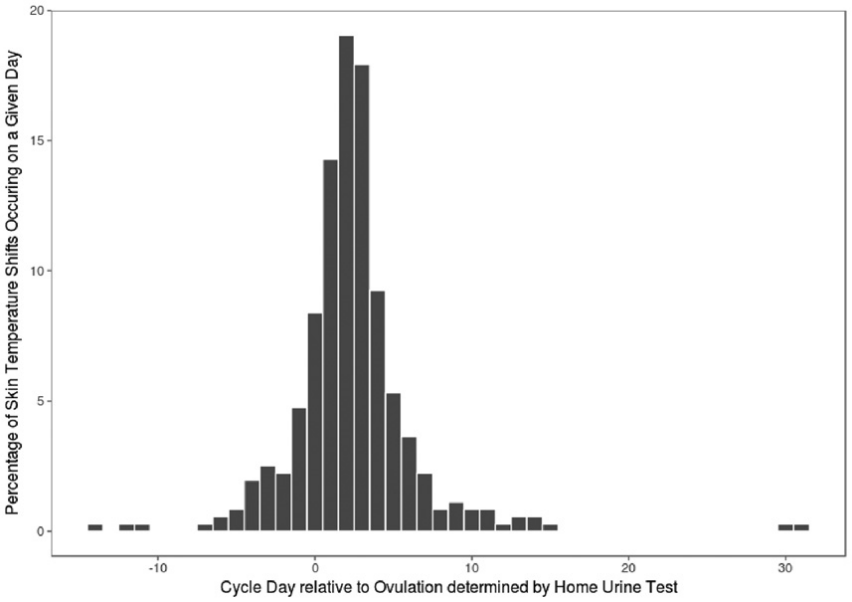
A histogram of the percentage of temperature shifts occurring on a given day with reference to home LH test (*n*=307)

We detected a shift in skin temperature in 82% of the cycles. The majority of detected temperature shifts (86%) took place on or after the OV (Figure 1). The choice of threshold at which the temperature shift occurred altered the aforementioned numbers minimally (e.g. setting the temperature threshold at 0.15°C yields 88 and 84%, respectively). None of the participants had exclusively monophasic temperature patterns nor temperature shifts occurring exclusively before ovulation (for participants with more than one cycle).

**Figure 2:**
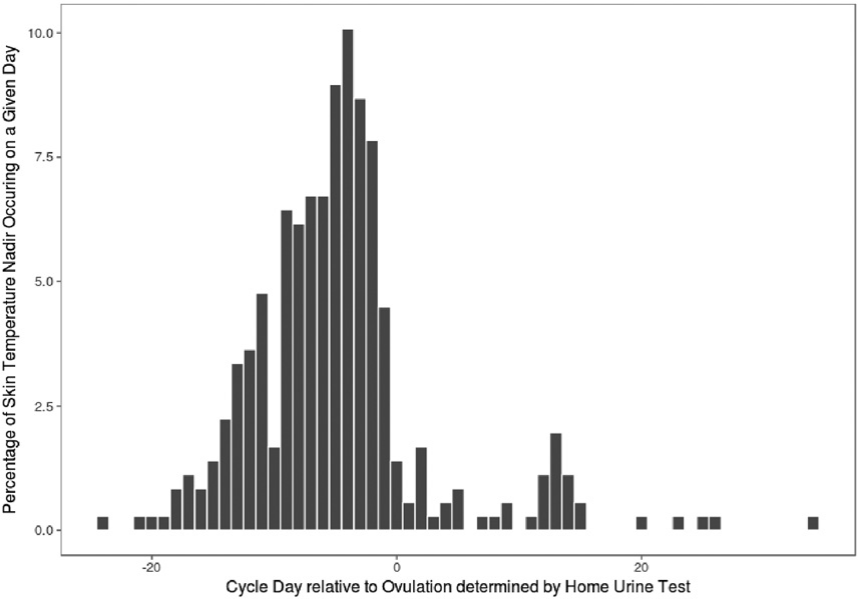
A histogram of the percentage of days on which the lowest temperature was observed in a given cycle with reference to home LH test (*n*=307)

Occurring mostly prior to ovulation, the lowest temperature in a given cycle was often observed outside the fertile window. Only in 41% of the 357 cycles was the nadir detected within the fertile window (OV-5 to OV; see Figure 2). Twelve percent of cycles showed a WST nadir occurring after ovulation, while the remaining 47% had the lowest WST reading prior to the fertile window. In sum, the majority (88%) of the biphasic cycles in our study exhibited a WST nadir prior to ovulation.

### Quantitation of the change in skin temperature across the menstrual phases

Consistent with our hypothesis and patterns in traditional BBT tracking, average WST during the menstrual phase (statistical mean (M) = 35.32°C, S.D. = 0.71) was significantly lower than the average WST during the early-luteal (M = 36.04°C, S.D. = 0.69; t(4.05) = 10.53, *P*<0.001) and late-luteal phases (M = 35.70°C, S.D. = 0.63; t(3.83) = 12.37, *P*<0.001). In addition, women had significantly lower WST (M = 35.23°C, S.D. = 0.67), on average, in their fertile phase compared with their menstrual phase WST (see Table 1).

### Influence of BBT-documented covariates on WST

Whereas prior research has found BBT readings and temperature shifts can be skewed by environmental factors [11,51,53,57–59], our findings suggest WST is robust to these confounders. For each covariate, we ran a separate multivariate, multilevel model predicting temperature in the 99th percentile based off phase and that covariate. The main effects of phase shift described above remained significant, even when controlling for covariates. Spotting, age, and having coffee or exercising in the 3 h preceding sleep did not significantly affect WST (see Table 2). Although a woman with a higher BMI was significantly more likely to have a lower WST, the direction and magnitude of menstrual phase shifts on WST remains unchanged. Having sex and eating a large meal in the 3 h before bed were also significantly associated with increases in nightly WST; however, the effect of menstrual phase on WST remained significant. Finally, the effect of the menstrual cycle on WST was significant even when controlling for drinking five or more units up to 3 h before bed. For the full model statistics, please see Table 2.

**Table 2:** Multilevel, linear mixed models of the relationship between menstrual phase, covariates, and sleeping WST

	Model 1	Model 2	Model 3	Model 4	Model 5	Model 6	Model 7	Model 8
**Cycle phase**								
Menstrual	Reference	Reference	Reference	Reference	Reference	Reference	Reference	Reference
Follicular	−0.03 (0.03)	−0.01 (0.03)	−0.03 (0.03)	−0.02 (0.00)	−0.03 (0.03)	−0.03 (0.03)	−0.03 (0.03)	−0.03 (0.03)
Fertile	−0.13§ (0.02)	−0.12§ (0.01)	−0.13§ (0.02)	0.13§ (0.00)	−0.12§ (0.01)	−0.13§ (0.02)	−0.13§ (0.02)	−0.13§ (0.02)
Early-luteal	0.24§ (0.02)	0.24§ (0.02)	0.24§ (0.02)	0.24§ (0.00)	0.24§ (0.02)	0.24§ (0.02)	0.24§ (0.02)	0.23§ (0.02)
Late-luteal	0.37§ (0.03)	0.38§ (0.03)	0.37§ (0.03)	0.37§ (0.00)	0.37§ (0.01)	0.37§ (0.03)	0.37§ (0.03)	0.37§ (0.03)
**Meal**^1^								
Small or no food	Reference							
Medium-sized	−0.02* (0.01)							
Large	−0.03‡ (0.01)							
**Body mass index (kg/m^2^)**		−0.05§ (0.01)						
**Coffee**^1^								
No coffee			Reference					
> =1			−0.01 (0.01)					
**Exercise**^1^								
No exercise				Reference				
<60 min				0.01 (0.01)				
>60 min				0.02 (0.01)				
**Sexual intercourse**^1^					−2.30† (0.01)			
**Alcohol**^1^								
No alcohol						Reference		
1–4 units						0.00 (0.01)		
≥5 units						0.06‡ (0.02)		
**Age (years)**							0.02 (0.01)	
**Spotting**								−0.01 (0.02)

Unstandardized b-coefficient values reported with S.E.M. in parentheses.

1 Within 3 h preceding the onset of sleep; *, †, ‡, § refer to *P*<0.10, 0.05, 0.01, 0.001, respectively.

Finally, we entered all the significant covariates into a full model with phase effects to further ensure the robustness of our hypothesis. As before, WSTs in the early- and late-luteal phases were significantly higher than WST in the menstrual phase, over and above the effects of any potential covariates (see Table 3). Our findings suggest biphasic shifts in WST across the menstrual cycle are detectable regardless of individual behavior or activities prior to sleep, a marked difference from potential confounds plaguing traditional BBT readings.

**Table 3:** Multilevel, linear mixed model of the relationship between menstrual phase and sleeping WST, controlling for all significant covariates

	Unstandardized b-coefficient	S.E.M.
**Intercept**	36.29	0.34
**Cycle phase**		
Menstrual	Reference	Reference
Follicular	−0.01	0.03
Fertile	−0.11§	0.01
Early-luteal	0.25§	0.02
Late-luteal	0.38§	0.03
**Meal**^1^		
Small or no food	Reference	Reference
Medium-sized	−0.02*	0.01
Large	−0.04§	0.01
**Body mass index (kg/m^2^)**	−0.05§	0.01
**Sexual intercourse**^1^	−0.02†	0.01
**Alcohol**^1^		
No alcohol	Reference	Reference
1–4 units	0.01	0.01
≥5 units	0.07§	0.02

*, †, § refer to *P*<0.10, 0.05, 0.001, respectively.

1 Within 3 h preceding the onset of sleep.

## Discussion

In the present study, a biphasic skin temperature pattern was identified in 82% of cycles. In addition, the nadir skin temperature did not provide a robust prospective estimation of ovulation. We observed the utility of the skin temperature as a retrospective confirmatory marker for ovulation in 86% of the cycles where a biphasic pattern was observed. Finally, we observed the robustness of WST measurements to environmental factors, including alcohol, coffee, exercise, food, and sex.

Our findings are consistent with evidence from traditional BBT studies, suggesting wrist temperature measurements tap into the same underlying mechanism. The occurrence of a monophasic, temperature pattern in ovulatory cycles in 18% of the regular ovulatory cycles corresponds to the range (0–20%) found in other studies that confirm ovulation via serum hormonal levels, ultrasound, or pregnancy [11,51,53,57–59]. In our study, the proportion of cycles with retrospective ovulation confirmation by virtue of a temperature shift matches those of earlier BBT studies [22,30]. Furthermore, the magnitude of the observed temperature shift in our study (${\bar{\Delta WST}}_{menstrual\;-\;early\;luteal}\;=\;0.33{}^{\circ}\text{C}$) falls within the observed range (0.28–0.56°C) seen in classical BBT studies [11,51,59,60]. Finally, the range of days in which the shift can occur varied broadly for both the present study and previous studies [51,53,54].

The BBT nadir is reported to be indicative of the OV and has been identified within 1 day of ovulation in 33–75% of the cycles in prior studies [61]. In this study, a nadir was identified in 41% of the cycles within the fertile window. Although seemingly low, it is not inconsistent with the variability of BBT nadirs found in prior studies, with temperature shifts occurring as early as 8 days prior to and 4 days post ovulation [11]. BBT nadirs have been found to differ from LH peaks in 55–70% of cycles [62,63]. While the initial rise in progesterone levels has been shown to occur consistently around ovulation and almost never earlier than a day before ovulation, estrogen levels are much more variable [64]. This could explain the more consistent observation regarding temperature elevation (associated with progesterone rise) and much less consistently the temperature decrease (associated with estrogen rise).

Although we estimated ovulation’s occurrence via at home LH tests, we did not confirm it in-lab via reproductive hormone levels or ultrasound. The number of cycles with a monophasic pattern might be due to: (i) a false positive urine LH test; (ii) misinterpretation or non-compliance of the user; or (iii) no (detected) shift in WST despite ovulation. We excluded 170 cycles out of 793 total (21%) due to no user-confirmed LH peak. This could hint toward user compliance issues with home urine tests, misuse of the device, unreliable monitors, or insufficient hormonal variation due to health conditions [33]. Apart from ultrasound, currently there are no consensus on the biomarkers for anovulation. The hormonal profile that constitutes an anovulatory cycle remains disputable, and the algorithms employed give varying rates of anovulatory cycles (3–19%; [65]). A false positive rate remains an open point for future research with ultrasound verification of ovulation as a reference.

One of the strengths of the skin temperature method using a wearable device compared with the oral, vaginal, or rectal BBT method is the continuous measurement during the night and the ease of providing automatic detection of the shift by computer programs or smartphone applications (e.g. cycle tracking applications). This renders the measurements less susceptible to measurement errors (e.g. different waking times) and misinterpretation of the results. Similar to other wearables measuring physiological parameters, the device used in the present study requires the user to sync it to the phone application. We excluded data from 186 cycles in our study due to user non-compliance on 20% of the days in a given cycle (e.g. not syncing 6 days out of a 28-day cycle); it is conceivable that this compliance issue could decrease or increase in the general population depending on the device used to measure WST (e.g. one would expect the compliance to increase if the device were to automatically sync once wireless internet or a Bluetooth connection is available without the user’s involvement). In the present study, the individuals were required to measure LH using urine tests, fill in a daily survey, and they were blind to the measurement of the device. It is plausible that in the real-world scenarios observing the daily progression of the physiological parameters and predictions would motivate the user for further compliance. Other current methods of fertility tracking are not impervious to similar limitations. Women who do not take their BBT measurements at precisely the same time daily or who miss several days in a given cycle may less easily detect a biphasic temperature shift [22]. Similarly, women using LH tests who skip a day of urinary testing run the risk of missing the only indication of a LH surge and impending ovulation [33]. Thus, it remains an empirical question for future studies whether women would have greater compliance using a less-invasive method like WST than using LH testing or traditional BBT.

On a related note, future studies may also consider whether users find WST fertility tracking less burdensome and inconvenient than BBT. Traditional BBT methods require women wake up several hours before rise to take their temperature [21]. Indeed, when asked about the burden associated with BBT methods, most women in a prior study found the method cumbersome; only 15% of patients described BBT procedure as having ‘no burden’ [53]. Because wearable devices, like the one used in our study, measure WST continuously throughout the night, they alleviate the need for users to wake up early. It follows then that they may be perceived by users as being less burdensome and/or more convenient. However, this is an empirical question and one that remains to be tested. It may be that the need to sync the device with a phone daily proves similarly burdensome as taking one’s vaginal, oral, or rectal temperature daily. Future studies could improve upon our findings, by exploring the potential ease of use and convenience that WST may offer over traditional BBT fertility tracking.

A second novel strength of wrist-based skin temperature measurement in fertility tracking appears to be its imperviousness to environmental factors. We show that wrist-based wearables are sensitive enough to pick up phase-based shifts in temperature, over and above any changes that may be due to having sex, food, or alcohol consumption. This presents an advancement in fertility awareness temperature tracking, allowing women increased accuracy in ovulation prediction without having to change prebedtime habits.

Ovulation tracking methods currently cost from ten to several hundred American dollars, depending on the underlying technology [22]. The calendar and BBT methods represent the lower spectrum of this price range while multiparameter devices (such as the one employed in the present study) and digital estrogen/LH measuring devices occupy the upper range. Each of the mentioned methods has advantages and disadvantages in terms of cost, convenience, being prospective, and accuracy. Further studies comparing current methods on the market will aid the user in deciding which of these methods offers a compelling case for their needs.

In addition to being reusable, multiparameter devices may also offer women insight into more of their fertile days than OPKs [46,66]. Although OPKs can prospectively predict the fertile window [21,22], they provide only a 12–48 h advanced notice that ovulation may be about to occur [21]. Multiparameter devices have the potential to prospectively predict more of the fertile days [46,66]. While the current study examined the confirmatory presence of a shift in WST over the fertile window, when paired with more physiological parameters (e.g. pulse rate [46]) and more sophisticated statistical methods (e.g. machine-learning), devices measuring multi-physiological parameters can learn an individual woman’s typical cycle physiological variation and use that to detect more of her fertile days. Furthermore, this information can be made easily readable and available via a phone application. This technology could be combined with OPKs for further tuning. For example, the application would indicate the approach of the fertile window, allowing women to better predict when to begin urinary LH testing. Using both methods together, women could better identify the highest fertility days (missed by LH testing alone) and confirm that ovulation took place. Combining fertility awareness methods is widely seen as the most effective way to track fertility, with numerous researchers suggesting incorporating newer technological advances to better identify the full fertile window [22,67,68]. Future studies should explore the successful conception rates of WST devices used in conjunction with other methods; it may be that this technological advance on traditional BBT provides the prospective window necessary to take advantage of the highest fertile days.

## Conclusion

In the present study, we demonstrated that the rhythm of skin temperature during menstrual cycle as measured by a wrist-worn wearable shows a biphasic pattern in 82% of the cycles, which is comparable with the results obtained in prior studies using BBT. Consequently, daily skin temperature measurements taken during the night with today’s wearable sensor technology could be a potential alternative for the oral, rectal, or vaginal BBT method. However, by itself and in agreement with the BBT method, WST did not capture all ovulation events and majorly, retrospectively. Therefore, skin temperature could be a potentially useful parameter to be combined with other physiological measurements correlating with the onset of the fertile window and with ovulation for a more comprehensive modern fertility awareness method.

## Clinical perspectives

- The present study investigates an alternative, more automatic method to traditional BBT monitoring, examining how WST changes across the menstrual cycle for eumenorrheic, non-pregnant women.
- We observe a similar shift in WST around the time of ovulation and the fertile window in the majority of participants, mirroring previously noted BBT shifts. Furthermore, whereas BBT can be prone to measurement error caused by daily activities (e.g. exercising or having a large meal prior to bed), we demonstrate WST’s imperviousness to these factors.
- Our findings suggest that WST could be a promising parameter for future multiparameter fertility-awareness methods. Easy to use and less invasive than oral, vaginal, or rectal BBT, wrist-worn wearables may empower users to become better attuned to their reproductive health and more readily identify phase-based changes in fertility-related biomarkers across the menstrual cycle.

## Footnotes

i Temprdrop Ltd, Hata’asiya 11 (Hubanana Hub), Ra’anana 43656, Israel (http://tempdrop.xyz/)

ii Ava AG, Räffelstrasse 26, Zurich 8045, Switzerland (http://www.avawomen.com/)

iii YONO Health Inc., 4500 Great America PKWY #1038, Santa Clara, CA 95054, U.S.A. (https://www.yonolabs.com/)

## Abbreviations

BBT: basal body temperature
BMI: body mass index
LH: luteinizing hormone
OPK: ovulation predictor kit
OV: ovulation day
WST: wrist skin temperature
